# Antibacterial Properties and pH Sensitive Swelling of Insitu Formed Silver-Curcumin Nanocomposite Based Chitosan Hydrogel

**DOI:** 10.3390/polym12112451

**Published:** 2020-10-23

**Authors:** M. M. Abd El-Hady, S. El-Sayed Saeed

**Affiliations:** 1 Department of Physics, College of Science and Arts, Qassim University, Al Asyah, P.O. Box 6666, Buraidah 51452, Saudi Arabia; 2 National Research Centre, Textile Research Division, 33 El-Behoth Street, Dokki, P.O. Box 12622, Cairo 11461, Egypt; 3 Department of Chemistry, College of Science, Qassim University, Buraidah, Saudi Arabia P.O. Box 6666, Buraidah 51452, Saudi Arabia; s.saeed@qu.edu.sa

**Keywords:** curcumin/silver nanoparticles, chitosan hydrogel, antibacterial properties, nanocomposite, swelling, pH sensitivity

## Abstract

A simple method was used to prepare curcumin/silver nanocomposite based chitosan hydrogel. In an alkaline medium, chitosan and chitosan nanocomposite hydrogels were prepared using the physical crosslinking method. The prepared hydrogels were stable for a long period at room temperature. In one step, silver nanoparticles were prepared insitu using silver nitrate solution and curcumin oxide within the hydrogel network formation. In the meantime, curcumin compound served as both a reducing and stabilizing agent. The structure and surface morphology of nanocomposite hydrogels were characterized by FTIR, SEM, and EDX analysis confirmed the formation of silver nanoparticles within the hydrogel network. Moreover, Images of TEM showed a spherical shape of silver nanoparticles with an average size of 2–10 nm within the matrix of the hydrogel. The formation mechanism of nanocomposite based hydrogel was reported. Besides that, the effect of chitosan and silver nitrate concentrations were studied. The swelling capacity of the prepared nanocomposite hydrogels was also performed at different pH of 4, 7, and 9. From the experimental results, the swelling capacity of hydrogels depends on the concentrations of chitosan and silver nitrate. The prepared composite based hydrogel exceeds a higher swelling degree than chitosan hydrogels at low pH. The antibacterial activity of the nanocomposite hydrogels was also examined; the results showed that the prepared nanocomposite hydrogels outperformed the pure chitosan hydrogels. This shows them to be a promising material for the biomedical field as a wound dressing and drug release.

## 1. Introduction

Hydrogels are crosslinked network structures with three-dimensional, polymeric, hydrophilic, which can absorb a large amount of water within their structures compared to other absorbing materials [1,2,3]. They are insoluble in water under physiological conditions. In addition, they can afford free-space within their network structure in the swollen form that helps for the growth and nucleation of nanoparticles [4]. As a result of its hydrophobic insoluble structure, it can be used in wound dressing applications. Also, they are formed either by physical, electrostatic, or chemical cross-links between polymer chains [5]. Hydrogels based on polysaccharide have attracted considerable interest and have been widely used as wound dressing because of their non-toxic, biocompatible, renewable, and biodegradable features [6]. In addition, hydrogels used as wound dressing materials have unique properties for enhancing the healing process. It should be used to fight bacteria, biocompatibility, and the high adsorption ability of fluids [7]. The growth of microorganisms on hydrogel causes problems in the biomedical field, so incorporation of antimicrobial agents is required. Among them, metal nanoparticles are characterized by fascinating properties such as magnetic, electrical, and antimicrobial activities, which cannot be detected by their bulk counterparts [8]. Chitosan was a natural cationic polymer that has many commercial and medical applications. In addition to the biomedical applications of chitosan, it can be used as an antibacterial agent. Chitosan is easily dissolved in diverse acids and converted to hydrogel or complexes [9]. Physically crosslinked chitosan hydrogels are a more popular technique than others, which are fabricated via chemical techniques [10], since they prevent possible toxicity and loss of intrinsic properties induced by crosslinking or reinforcement [11]. These physically crosslinked hydrogels may also be used in the manufacture of chitosan composite substances. They also might be formed by the addition of other materials (such as metal ions or some reinforcements) [10]. Silver nanoparticles (AgNPs) have unique properties such as biological and catalytic properties. Several methods for the preparation of silver nanoparticles are developed because of its broad-spectrum of a variety application such as clothing, the food industry, and optics [12,13]. Controlling the size, shape and less of aggregation is the most efficient method for the preparation of silver nanoparticles [14]. Recently, silver nanoparticles have been incorporated in hydrogels and polymers for medical applications [15]. The incorporation of silver nanoparticles into hydrogels has been shown to modify gelation and antibacterial activity [16,17,18,19]. Curcumin is a hydrophobic poly phenolic compound exhibiting powerful biological activities [19]. Antibacterial properties and tissue repair efficiency of curcumin compound have been proved by several researchers [20]. However, it has poor solubility in water, restricting its application in the biomedical field. To overcome this problem, curcumin can form composites with Chitosan. Previous reports investigated pH sensitivity [21]; wound healing [22] and drug release [23] of loading curcumin compound based chitosan hydrogel. Recently, chitosan hydrogel was developed by incorporating curcumin complexed with beta cyclodextrins as a wound dressing [24]. Others used curcumin–gelatin fabricated chitosan hydrogel for wound healing [23]. Considering the antibacterial applications of chitosan, silver nanoparticles and curcumin, their composite combination may be effective against a wide range of bacteria as a wound dressing.

In this work, we fabricate silver nanoparticles using curcumin, a polyphenol compound, as both a reductant and a stabilizing agent. In addition, the curcumin compound improved the efficiency of antibacterial activity within prepared nanocomposite hydrogels. We also reported the development of a chitosan-based hydrogel using a physical crosslinking technique to investigate the addition of curcumin as a reducing agent for in-situ preparation of silver nanoparticles during the formation of hydrogel. The novelty of our work, preparation of chitosan/silver-curcumin nanocomposite hydrogel by a green, cost-effective and eco-friendly method. The prepared nanocomposite hydrogel is a candidate for wound dressing applications. The effect of chitosan and silver nitrate concentrations on the properties (swelling and pH sensitivity) of nanocomposite hydrogels was discussed. The antibacterial activity of the hydrogels was also investigated.

## 2. Materials and Methods

### 2.1. Chemicals

Chitosan high molecular weight Mw 310,000–375,000 Da, deacetylation degree ~85%, 800–2000 cP, acetic acid, ethanol, sodium hydroxide, and curcumin powder (99.8% pure and anhydrous) was purchased from Sigma-Aldrich (Taufkirchen, Germany). Silver nitrate (AgNO_3_) we purchased from Merck.

### 2.2. Preparation of Chitosan Hydrogel

2.0 g of chitosan was dissolved in 0.05 M acetic acid (100 mL). A sodium hydroxide solution was then slowly added to the chitosan solution until the pH reached 8.5–9.0. The obtained hydrogel was decanted and washed multiple times using distilled water. Finally, the hydrogel was dried overnight and was stored until further study.

### 2.3. Preparation of Chitosan/Silver–Curcumin Nanocomposite Hydrogel 

Chitosan was initially dissolved in 1% acetic acid at different concentrations (1%, 2% and 3%) and later stirred on a hot plate for two hours at 25 °C at 500 rpm to facilitate its dissolution. 30 mL of chitosan solutions were added to 5 mL of aqueous solution of AgNO_3_ at different concentrations (0.5%, 1%, and 1.5%). Then, the mixed solutions were stirred thoroughly for 10 min via magnetic stirring. 0.1 g of curcumin powder into NaOH (1 M) solution to form curcumin oxide solutions. 0.75 mL of curcumin oxide solution was added drop-wise into chitosan silver nitrate solutions. The mixed solutions were stirred thoroughly for 2 h in the dark at 25 °C to the complete formation of a hydrogel. The color of the solutions changed immediately from light yellow to brownish yellow, showing the formation of Ag NPs. The obtained hydrogel was washed several times with distilled water for removing extra-unreacted silver ions and sodium hydroxide. Finally, the hydrogel dried overnight for further investigation.

### 2.4. Characterization

#### 2.4.1. Swelling Properties of Hydrogels 

The swelling degree of prepared hydrogels was determined via soaking 0.1 g of a dried hydrogel in 50 mL of the medium at 37 °C. Then we extended hydrogel swelling in the distilled water, acidic, and basic medium by varying pH conditions using buffer solutions of pH 4, 7, and 9. Moreover, the weight of swollen hydrogel was also measured at time intervals 15, 30, 60, 120, and 180 min. The swelling degree at equilibrium was calculated using Equation (1).

(1) $$swelling\;degree\;\left(SD\right)=\frac{Ws-Wd}{Wd}\;\times 100\;$$

where *W_d_* is the dry hydrogel weight (g) and *Ws* is the swollen hydrogel weight (g)

#### 2.4.2. Fourier-Transformed Infrared Spectroscopy (FT-IR)

FTIR was recorded on a ATR-FTIR instrument (JASCO, Model IR 4700, Tokyo, Japan) and scanned from 4000 to 400 cm^−1^ in ATR mode using KBr as supporting material.

#### 2.4.3. Scanning Electron Micrograph SEM/EDX Analysis

Samples for SEM/EDX were taken using FEI INSPECTS Company, Philips, Eindhoven, Holland environmental scanning without coating. Elemental micro-probe and elemental distribution mapping techniques were used for analyzing the elemental constitution of solid samples. Elemental analysis of the particles was implemented by SEM equipped with an energy dispersive spectroscope (EDX), to get rapid quantitative and qualitative analysis of the elemental composition.

#### 2.4.4. Transmission Electron Microscope (TEM) Analysis

Transmission electron microscopy (TEM) was used to determine the size of silver nanoparticles inside the hydrogel. The dried hydrogels were grounded with the help of a softball and the resulted hydrogel containing silver nanoparticles was dispersed in 1 mL of distilled water for 3 days to extract the silver nanoparticles from the hydrogel network into the aqueous phase. As per our observations, this grinding process was highly efficient to determine the size of silver nanoparticles in the hydrogel, since there is no change in the size of nanoparticles.

#### 2.4.5. Antibacterial Test

Antimicrobial activity of the prepared hydrogel was evaluated using the agar diffusion test according to AATCC Standard Test Method 147–1988. A mixture of nutrient broth and nutrient agar in 1 L distilled water at pH 7.2, as well as the empty Petri plates, were autoclaved. The agar medium was then cast into the Petri plates and cooled in laminar airflow. Approximately 105 colony-forming units of bacteria were inoculated on plates, and then hydrogel samples were planted onto the agar plates. All the plates were incubated at 37 °C for 24 h and examined if a zone of inhibition was produced around samples.

#### 2.4.6. Statistical Analysis

Results were expressed as a mean value with its standard deviation (mean ± S.D.) of each sample, which was repeated three times (n = 3). 

## 3. Results and Discussion

### 3.1. Mechanism of Prepared Hydrogel

**Figure 1:**
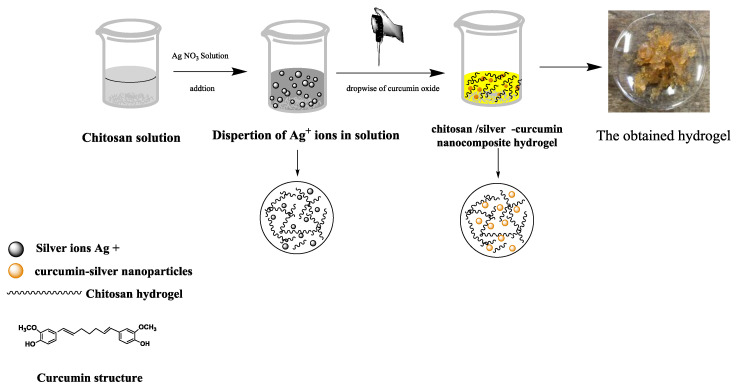
Schematic diagram for the synthesis of chitosan/silver -curcumin nanocomposite hydrogel.

The importance of chitosan–silver nanoparticles hydrogels are very necessary in the biomedical field. For this reason, the hydrogels of chitosan embedded silver nanoparticles can be synthesized easily from a chitosan solution using physical crosslinking in a sodium hydroxide solution [25]. In addition, silver-curcumin nanoparticles were prepared inside the hydrogel network during its formation. Moreover, this study depends on the dual function of a curcumin molecule in the form of curcumin oxide as a reducing agent for silver nanoparticles and serves in the formation of a chitosan hydrogel. Figure 1 shows the schematic diagram for the synthesis of a chitosan/silver-curcumin nanocomposite hydrogel. The use of chitosan allowed the Ag^+^ ions to interact and form complex or non-covalent interactions with free hydroxyl (–OH) and amine (–NH_2_) functional groups of chitosan polymeric chains. Then the addition of a dropwise curcumin oxide solution resulted in a combination of curcumin with Ag^+^ ions to form [Ag (curcumin)]^+^ complex. The obtained complex further reacted with an aldehyde group (from the ethanoic group of curcumin chemical structure) to form [Ag (curcumin)] through the reduction of Ag^+^ ions. The formation of nano [Ag (curcumin)] particles and reduction of Ag^+^ ions were due to the oxidation of the aldehyde group into the carboxylic group [26]. In addition, there was an intramolecular hydrogen bond between curcumin and chitosan molecules. Hydroxyl oxygen on the benzene ring is an effective binding site for chitosan in a curcumin molecule. One hydrogen bond can be formed by free hydroxyl groups of glucosamine and the other can be formed by the free amino group of glucosamine [27], resulting in the formation of a hydrogel network with evenly distributed silver-curcumin nanoparticles.

### 3.2. Swelling Studies

**Figure 2:**
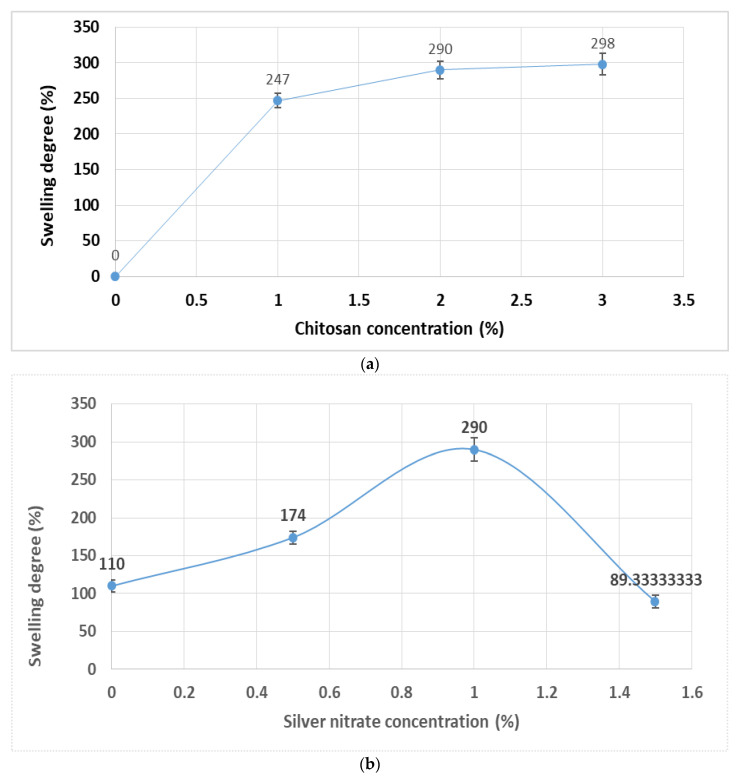
(**a**) effect of chitosan concentration on the swelling degree of the hydrogel, (**b**) effect of silver nitrate concentration on the swelling degree of the hydrogel.

The swelling degree of a synthesized hydrogel may be altered by either change in concentrations of chitosan, or silver nitrate [8]. Figure 2a shows the effect of chitosan concentration on the swelling degree of the hydrogel. It is clear that as the concentration of chitosan increases from 1% to 2%, the swelling degree increases from 247% to 290%. This is due to the availability of more free spaces in the gel. Further, increase in concentration of chitosan to 3% resulted in insignificant improvement in the swelling degree by 298%. While Figure 2b shows the effect of the concentration of silver nitrate on the swelling degree of the hydrogel, it is obvious that at zero concentration of silver nitrate the swelling degree is 110%. As the concentration of silver nitrate increases from 0.5% to 1.0%, the swelling degree of the hydrogel increases from 174% to 290%. Therefore, the degree of swelling increased in the hydrogels following the increased amount of [Ag (curcumin)] nanoparticles. Absorption of water in the nanocomposite structure increases as the increased content of nanoparticles prepared in the hydrogel structure leads to the development of pores in the hydrogel matrix [28]. In addition, because of the charged [Ag (curcumin)] nanoparticles, the molecules of water penetrated more for the osmotic pressure balance. Therefore, after the formulation of [Ag (curcumin) nanoparticles, the hydrogel network can be expanded and thus more pores and free spaces are required in the hydrogel network. Further increase in the concentration of silver nitrate from 1.0% to 1.5% resulted in a decrease in the swelling degree from 290% to 89.33%. This may be attributed to the likely formation of more cross-links between chitosan chains and the filling up of the free space of hydrogel by [Ag (curcumin)] nanoparticles. Consequently, the degree of swelling decreases [29]. From the above results, the proper concentrations and conditions for preparing chitosan/Ag-curcumin nanocomposite-based hydrogel are 2% chitosan, 1.5% silver nitrate solution, and pH 8.5–9.

### 3.3. pH-Sensitivity on Swelling Behavior of Chitosan and Chitosan/Ag–Curcumin Nanocomposite Hydrogels

**Figure 3:**
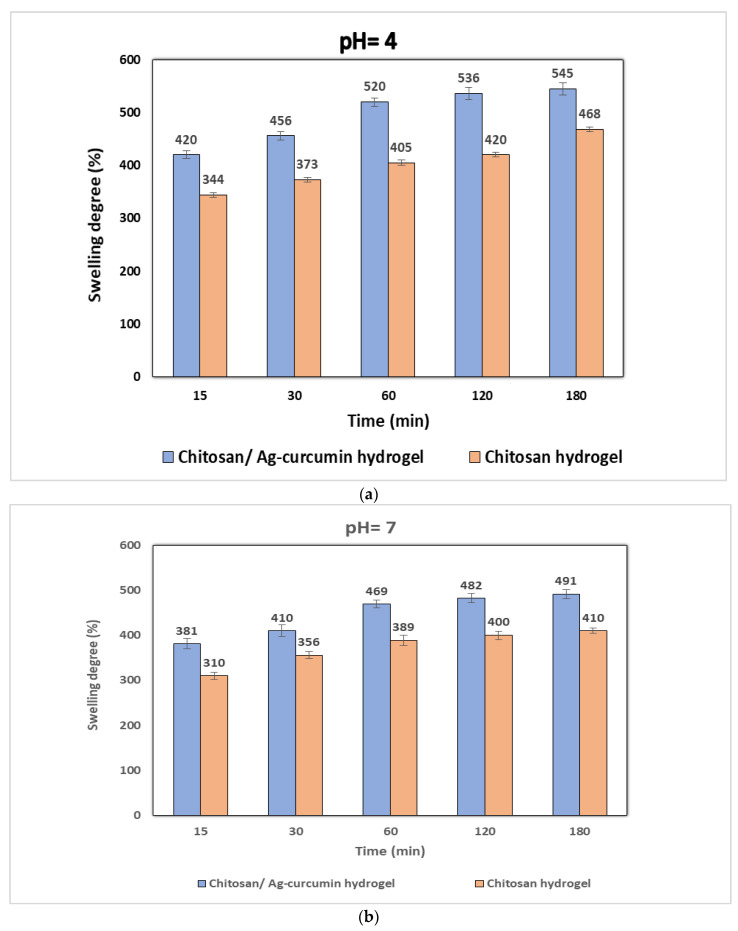

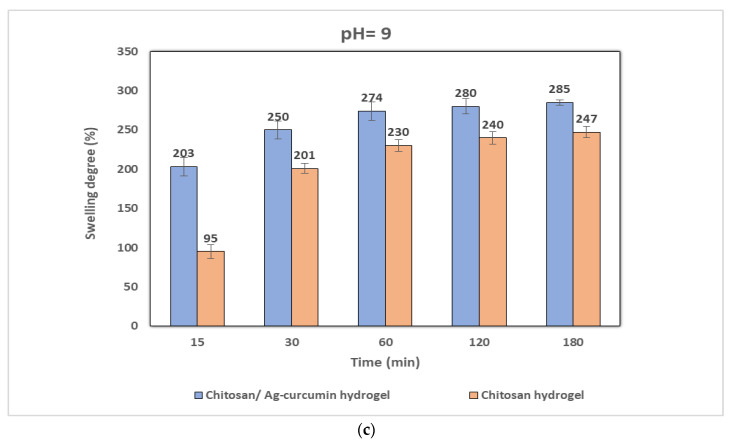
Swelling degree of hydrogel samples in respect of different (**a**) pH 4 (**b**) pH 7, and (**c**)pH 9.

To better understand the effects of chitosan hydrogel and chitosan/silver-curcumin nanocomposite hydrogels networks formed in response to pH 4, pH 7, and pH 9 buffer solutions at different times, several experiments were carried out at a fixed temperature 37 °C. Figure 3 shows the pH sensitivity on swelling behavior of chitosan and chitosan/Ag–curcumin hydrogels. It is obvious from Figure 3 that in the first 60 min the hydrogels displayed rapid swelling, which gradually reached an equilibrium value. Initial swelling is due to the hydrogen bond between the chitosan, chitosan/Ag–curcumin and water molecules functional groups. Moreover, a network-like structure was developed because of hydrogen bonds between the bound water molecules and more water molecules. Finally, higher swelling is caused by excess water, which enters the hydrogel’s network [30]. Maximum hydrogel swelling was observed at low pH. Additionally, the degree of swelling was decreased by pH increases. Moreover, the swelling was also low at a neutral pH, i.e., 7. The chitosan molecule has basic groups of amino and hydroxyl in acidic pH and is soluble in an acidic solution. The amino group (NH_2_) changed to NH_3_^+^ after dissolving chitosan in 1% acetic acid. Then, chitosan has mostly NH_3_^+^ as a functional pendate group. There may be excess positive charges during swelling at low pH that repel each other to provide the highest swelling. This could be explained by providing free spaces for molecules of water to enter the network of the hydrogel and generate hydrogen bonds. On the other hand, the lowest degree of swelling was observed at higher pH, since there are less protons available to produce a positive charge on the polymeric structure of hydrogels. Additionally, chitosan/Ag-curcumin hydrogel has a swelling degree higher than chitosan hydrogel. This may be because the presence of Ag-curcumin nanoparticles in the hydrogel could expand and the network of formed hydrogel also enhanced the free spaces within its matrix; consequently, chitosan/curcumin-Ag hydrogel adsorbs extra water [31]. Such composite-based hydrogels have pH-sensitive properties; they can be used as drug release at a certain pH in a controlled manner.

### 3.4. IR Analysis

**Figure 4:**
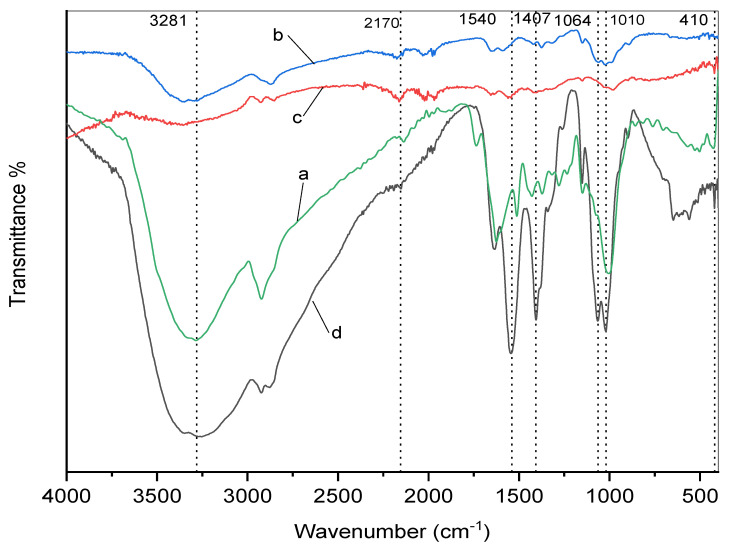
FTIR spectrum of (**a**) curcumin powder, (**b**) pure chitosan, (**c**) chitosan–Ag curcumin hydrogel, (**d**) chitosan hydrogel.

Figure 4 shows the FTIR spectra of curcumin (a) of pure chitosan (b) of chitosan/Ag –curcumin nanocomposite hydrogel (c) and of chitosan hydrogel (d). The spectrum of curcumin in Figure 4a shows an absorption peak at 3310 cm^−1^ indicated to the stretching vibration of phenolic O-H. In addition, sharp absorption peaks in the region range from 1450 to 1630 cm^−1^. These peaks correspond respectively to groups –OH, C=O, and C=C (enol). Other peaks in the region between 1000 cm^−1^ and 1300 cm^−1^ were observed. All peaks are ascribed to the configuration of the symmetric and asymmetric C–O–C groups [32]. The spectrum of pure chitosan in Figure 4b shows the characteristic peak at 3347 cm^−1^ can be attributed to –NH_2_ and-OH groups stretching vibrations. The peak at 1637 cm^−1^ refers to amide carboxyl group. Other peaks from 1000 to 1100 cm^−1^ can be attributed to three differential ring vibrations of C–O–C, C–OH, and C–C [33]. The spectrum of chitosan/Ag-curcumin hydrogel in Figure 4c shows the presence of weak intensity peaks for chitosan and curcumin in chitosan/Ag- curcumin hydrogel spectrum. The incorporation of curcumin into chitosan broadened the peaks. In comparison to Figure 4a,b, there is a new sharp peak at 2170 cm^−1^. This may be attributable to the intense hydrogen bonding between the curcumin with either silver ions or chitosan chains [22]. Moreover, there is a peak at 410 cm^−1^ related to the formation of silver nanoparticles [27]. On the other hand, the chitosan hydrogel spectrum in Figure 4d shows a broad band at 3281 cm^−1^, corresponding to O–H stretching that overlaps the N-H stretching in the same region. While peaks at 1064 cm^−1^ and 1010 cm-1 are related to –C–O–C– glycosidic linkage in the chitosan ring. Other peaks at 1540 cm^−1^ and 1407 cm^−1^ are the alkyl and methyl groups of the C–H bending vibrations, respectively.

### 3.5. SEM Analysis

**Figure 5:**
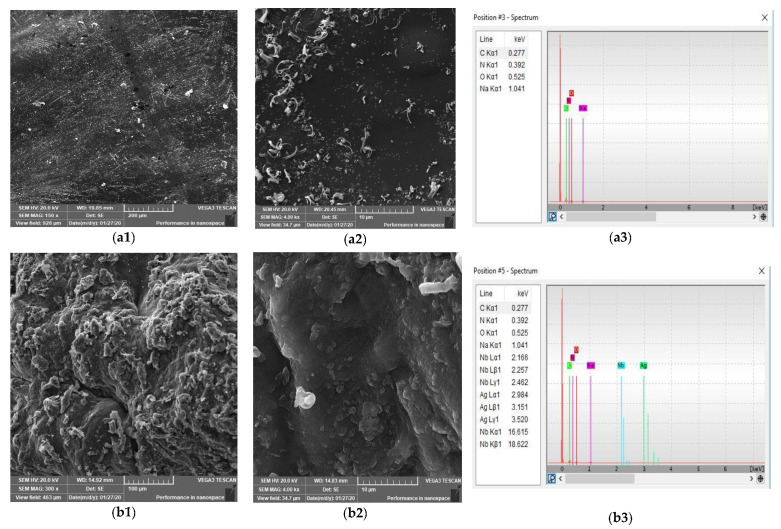
SEM images of chitosan hydrogel (**a1**, **a2**), chitosan/Ag –curcumin hydrogel (**b1**, **b2**) and their EDX spectra of chitosan hydrogel (**a3**) and chitosan/Ag-curcumin hydrogel (**b3**).

SEM images chitosan and chitosan/silver –curcumin hydrogels are depicted in Figure 5. In Figure 5(a1,a2), it has been observed that the surface of chitosan hydrogel is flat, clear, and uniform. Even so, some cracks were found on the surface of the chitosan hydrogel at certain places. While in Figure 5(b1,b2), on the surface of chitosan/Ag-curcumin nanocomposite hydrogel, silver nanoparticles could be clearly seen as white dots agglomerated at certain points by the presence and aggregation of curcumin particles. This was further confirmed by EDX analysis that shows peaks of C, O, N, Ag, and Nb of expected elements confirmed the formation of silver nanoparticles. 

**Figure 6:**
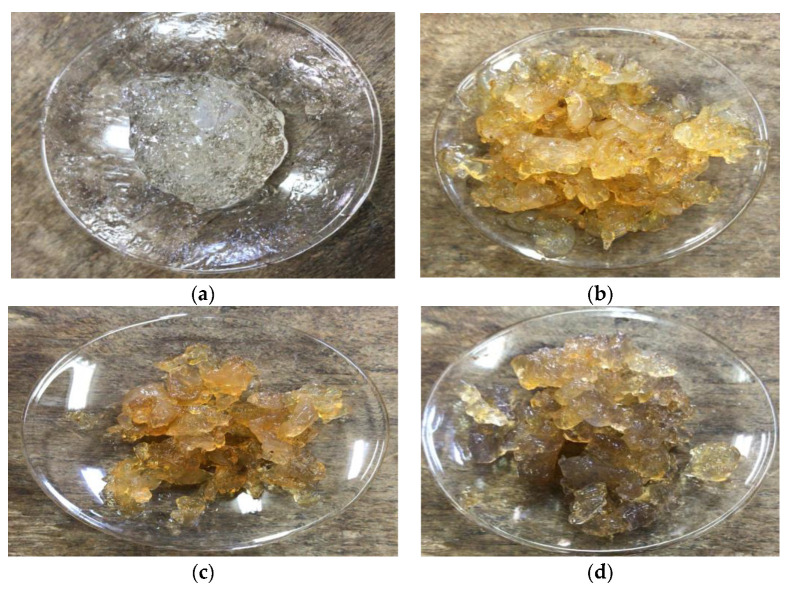
Digital photographs of prepared hydrogels. (**a**) Chitosan hydrogel; (**b**) 2% chitosan/0.5% Ag- curcumin nanocomposite hydrogel; (**c**) 2% chitosan/1% Ag-curcumin nanocomposite hydrogel; (**d**) 2% chitosan/1.5% Ag-curcumin nanocomposite hydrogel.

Color and shape in particular can potentially be used as a quick and simple way to easily detect hydrogels. The Digital (visual) photographs reflectance spectra are shown in Figure 6. Digital photographs reveal clear change in the color of chitosan hydrogel compared to chitosan/Ag-curcumin nanocomposite hydrogels. As expected, the introducing of [Ag-curcumin]^+^ particles in the chitosan solution was followed by a change in color from white to brownish-yellow. After reduction of Ag^+^ to form [Ag-curcumin] nanoparticles induced, hydrogel color change from yellow to yellowish-brown, by increasing concentration of the silver nitrate, confirmed the formation chitosan/Ag-curcumin nanocomposite hydrogel.

### 3.6. TEM Analysis

**Figure 7:**
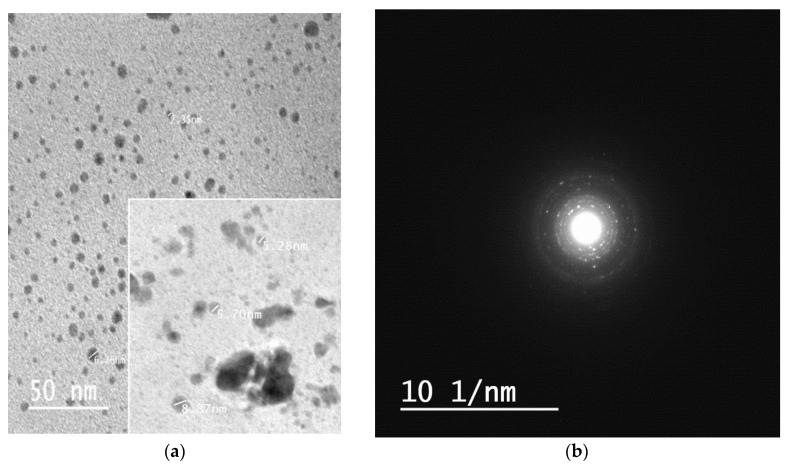
TEM and SEAD micrographs of prepared silver nanoparticles within chitosan/silver– curcumin hydrogel (**a**,**b**).

The size and shape of formed silver nanoparticles within the hydrogel matrix was examined using the TEM analysis. Figure 7a,b display the TEM and selected area electron diffraction (SAED) images. The particles are mainly spherical in shape with particle size varying between 2–10 nm with fewer aggregates. It is obvious from Figure 7 that the particles are separated from each other. These results reflect that curcumin oxide not only acts as a strong reducing agent for Ag^+^ but also has extra stabilizing action on silver nanoparticles formed within the matrix of chitosan/Ag-curcumin nanocomposite hydrogel [34]. Moreover, the spherical shape of silver nanoparticles may be due to the presence of phenolic hydroxyl groups in curcumin during hydrogel formation [35].

### 3.7. Antibacterial Activity

The antibacterial activity results of chitosan hydrogel and chitosan/silver-curcumin nanocomposite hydrogel as determined by the agar diffusion method are shown in Table 1. The results reveal that chitosan/silver-curcumin nanocomposite hydrogel has better antibacterial activity compared to chitosan hydrogel. The inhibition zone recorded for chitosan/silver-curcumin nanocomposite hydrogel against Gram-positive (*S. aureus* and *B. subtilis*) and Gram-negative (*E. coli* and *P. aeruginosa*) were higher in comparison to those of chitosan hydrogel. This is because of the synergic effect of in-situ formed silver nanocomposite hydrogel. As reported, silver nanoparticles can interact with the cell membrane via the moiety of silver ions and interrupt the bacterial cell wall’s electrical balance. After membrane destruction, DNA and RNA together with other cytoplasmic fluids are emitted, into the extracellular matrix prior to the death of the cell [31,36]. Also, the action effect of curcumin molecules act as both stabilizing and reducing agent resulting in a higher bactericidal effect. This is achieved by creating silver nanoparticles of smaller size with a greater surface area that can improve the performance to access the bacteria’s cell membrane to enhance antibacterial activity [37]. Moreover, all studies about the bactericidal effect of chitosan proved that positively charged amino groups capable of interacting with negatively charged cell membranes which interrupt regular membrane functions, therefore, promoting the leakage of proteins and other intracellular components [14]. On the other hand, both hydrogels are more effective towards the Gram-negative bacteria than the Gram-positive bacteria strains; this may be due to the differences in the structure of the bacterial cell wall organization. In addition to the fact that Gram-positive bacteria have a thicker layer cell than Gram-negative bacteria, it acts as a hindrance and protects the cell wall from the spread of active ingredient into the cytoplasm [38].

## 4. Conclusions

In summary, we succeeded in the preparation of a nanocomposite hydrogel based on chitosan, silver nanoparticles and chitosan. The prepared hydrogel was formed by a simple, green, and eco-friendly method. Silver nanoparticles in form of silver-curcumin nanoparticles were prepared inside the hydrogel network during its formation. It sensitized insitu using curcumin in an alkaline solution. TEM images show silver nanoparticles have a spherical shape with an average size of 2–10 nm. The swelling capacity of hydrogels depends on concentrations of chitosan and silver nitrate solutions. Moreover, prepared nanocomposite hydrogels showed pH sensitivity with the highest swelling degree at low pH. Also, it has a higher swelling degree compared to pure chitosan hydrogel. In addition, it exhibited higher antibacterial activity against Gram-negative bacteria than Gram-positive bacteria, which is promising for use in wound dressing applications. 

## Figures and Tables

**Table 1:** The antibacterial activity of chitosan hydrogel and chitosan/Ag- curcumin nanocomposite hydrogel.

Sample	Inhibition Zone Diameter (mm/mg Sample)			
	G^+^		G^−^	
	*Bacillus subtilis*	*Staphylococcus aureus*	*Escherichia coli*	*Pseudomonas aeruginosa*
Chitosan hydrogel	10	10	12	11
Chitosan/Ag-curcumin nanocomposite hydrogel	13	13	15	14
